# Effect of preoperative oral carbohydrates on insulin resistance in patients undergoing laparoscopic cholecystectomy: a randomized controlled trial

**DOI:** 10.1007/s00423-024-03268-1

**Published:** 2024-02-27

**Authors:** Xiaohan Wang, Jingwen Zhuang, Jianxin Cheng, Zeyang Wang, Jingyi Sheng, Shanshan Guo, Rui Wang, Zhiping Wang

**Affiliations:** 1 Xuzhou Medical University, Xuzhou, 221004 Jiangsu China; 2 Jiangsu Province Key Laboratory of Anesthesiology, Xuzhou Medical University, 209 Tongshan Road, Xuzhou, 221004 Jiangsu China; 3 Department of Anesthesiology, The Affiliated Hospital of Xuzhou Medical University, Xuzhou, 221002 Jiangsu China

**Keywords:** Oral carbohydrates, Insulin resistance, Gastric emptying, Cholecystectomy, Laparoscopy

## Abstract

**Purpose:**

The present research seeks to clarify the consequences of two specific preoperative oral carbohydrate (POC) amounts on insulin resistance (IR) and stomach evacuation in laparoscopic cholecystectomy (LC) patients.

**Methods:**

A total of 129 patients set for elective LC procedures were randomly assigned to a control group (C, *n* = 45), a 200 mL POC group (P1, *n* = 42), and a 400 mL POC group (P2, *n* = 42). The C group was fasted from midnight until surgery, whereas the P1 and P2 groups received their respective carbohydrate volumes 2–4 h before anesthesia. Fasting blood glucose, insulin, and glucagon concentrations were measured at three junctures. IR metrics were derived by employing the homeostasis model assessment. Gastric volume was measured before anesthesia using gastric ultrasound. Inter-group comparisons included IR indicators, subjective comfort scores, and hemodynamic data.

**Results:**

At T2, the C group exhibited reduced glucose concentrations compared to the P2 group (4.73 ± 0.64 vs. 5.26 ± 1.02 mmol/L, *p* < 0.05). The Perlas grading indicated that grade 1 was more prevalent in the P2 group than in the P1 and C groups (18 [42.9%] vs. 6 [14.3%] and 1 [2.2%], *p* < 0.05). Additionally, thirst and hunger metrics for the P2 group were notably reduced compared to the C group at both T2 and T3.

**Conclusion:**

Administering either 200 mL or 400 mL of carbohydrates 2–4 h pre-surgery had no detectable impact on IR or gastric volume in LC patients.

**Trial registration:**

ChiCTR, ChiCTR2200065648. Registered January 13, 2023, http://www.chictr.org.cn.

## Introduction

Preoperative fasting primarily serves to mitigate the risk of reflux and aspiration during general anesthesia [1]. Traditionally, patients were required to abstain from solid food for 6 to 8 h and from liquids for 4 h before surgery [2]. However, patients typically ceased eating post-dinner, adhering to their usual dietary habits on the eve of surgery. Furthermore, unforeseen circumstances, such as the need to convert to laparotomy during endoscopic procedures, can unexpectedly prolong operation durations. This, in turn, leads to delays in initiating subsequent surgeries. Consequently, the actual duration of preoperative fasting and fluid restriction was often extended beyond the initially planned timeframes [3]. The combination of surgical stress and extended preoperative fasting accentuates the perioperative metabolic response and amplifies postoperative insulin resistance, effects linked to post-surgery complications and increased mortality [4]. Consequently, the exploration of reduced fasting durations has been the focus of much research.

Myriad studies have illuminated the benefits of preoperative oral carbohydrates (POCs) in alleviating adverse sequelae induced by prolonged fasting [5, 6]. Using POC before various elective surgeries is highly recommended by the Enhanced Recovery After Surgery (ERAS) Society, primarily to enhance insulin resistance and patient comfort [7, 8]. The use of preoperative drinks is acknowledged for its metabolic importance in extensive abdominal surgeries, which often lead to significant insulin resistance [9, 10]. However, the application of this intervention in procedures with smaller stress responses, like laparoscopic cholecystectomy, remains less understood.

Gallstones are prevalent in the digestive tract, with laparoscopic cholecystectomy (LC) emerging as the primary therapeutic intervention. Intriguingly, despite strict preoperative fasting, approximately 13% of patients with cholelithiasis still exhibit satiety before surgery [11], which is postulated to be due to impaired gastrointestinal peristalsis caused by gallstones and surrounding tissue inflammation [12]. Ultrasonography at the bedside is gaining traction as a method to determine gastric content type and quantity [13]. Recent studies have indicated that taking a carbohydrate beverage 2–4 h prior to anesthesia neither exacerbates the gastric volume (GV) [14] nor reduces the gastric pH value [15]. Therefore, POC is a safe and feasible option in clinical practice given the high reliability and accuracy of gastric ultrasound in evaluating the stomach contents [16].

Therefore, the present study’s aims were to clarify the impact of two distinct POC dosages on insulin resistance in individuals who underwent LC. Additionally, gastric ultrasound was used to measure stomach content volume to evaluate the potential likelihood of pulmonary aspiration.

## Materials and methods

### Study design and patients

A forward-looking randomized controlled trial was conducted at the Affiliated Hospital of Xuzhou Medical University. Our ethics committee approved the trial and its protocol (XYFY2021-KL083-01), which was registered with the Chinese Clinical Trial Registry (ChiCTR2100044373). The trial took place from December 2022 to June 2023 at the above hospital, a tertiary institution in Jiangsu, China. All patients provided written consent before taking part in the trial.

Patients who were scheduled to undergo elective LC were recruited with the inclusion criteria being: age 18–65 years, American Society of Anesthesiologists (ASA) physical status I–II, and a BMI 18–30 kg/m^2^. The exclusion criteria were gastric anatomic abnormalities or pathologies, a previous history of surgery on the esophagus or stomach, diabetes mellitus with unstable glucose concentrations or its clinical complications, pregnancy, and inability to communicate effectively with the researchers.

### Randomization, blinding, and intervention

Using a computer-generated code sequence through simple randomization, patients were evenly distributed into three groups: a control group (C group), a 200 mL oral carbohydrate group (P1 group), and a 400 mL oral carbohydrate group (P2 group). The sequence for randomization was kept hidden inside sealed, non-transparent envelopes. A nurse, who was not part of the data collection team, opened the envelopes in the ward and then provided the corresponding carbohydrate solution to the patients. On the day before surgery, all subjects were instructed to abstain from solids starting at 22:00 and from liquids after 24:00. Those in the P1 group were required to consume orally 200 mL of carbohydrates (200 mL/bottle, ShuNeng, carbohydrate 12.5 g/100 mL, energy 213 KJ/100 mL, and electrolytes; Humanwell, Yichang, China) 2–4 h before surgery within 5 min. Patients in the P2 group were required to intake orally 400 mL of the same solution 2–4 h before surgery within 10 min. Those in the C group were not allowed any food or drink on the day of their operation.

In our research, to ensure homogenization of management, we chose patients undergoing cholecystectomy at the first and second stations. On the surgery day, the surgeon and anesthesiologist closely communicated to determine the specific timing for carbohydrate consumption. Subsequently, the itinerant nurse called the ward nurse to provide the carbohydrates to the patients. Patients who drank fluids more than 4 h before surgery or less than 2 h apart were excluded. Patients with less than 2 h will delay the start time of surgery to a safe range. Both data collectors and statistical analysts were blinded to the grouping.

### Anesthesia

The procedures for general anesthesia and the operation were standardized and executed by a dedicated team of anesthesiologists and surgeons. Upon securing the necessary monitoring measures, such as non-invasive blood pressure, ECG, SpO_2_, temperature, and the BIS index, a sequential induction of general anesthesia was commenced. This entailed the intravenous administration of 0.05 mg/kg midazolam, 0.3 mg/kg etomidate, 0.3–0.5 μg/kg sufentanil, and 0.6 mg/kg rocuronium. Subsequent to the induction, volume-controlled mechanical ventilation was initiated following endotracheal intubation. The ventilatory parameters were adjusted to maintain a PetCO_2_ between 35 and 45 mmHg. Anesthesia was maintained using 1–2% inhaled sevoflurane, a continuous infusion of 0.2–0.5 μg/kg/min remifentanil, and 4–12 mg/kg/h propofol. Blood pressure and heart rate were stabilized with phenylephrine and atropine. BIS was maintained in the range of 40–60 by modulating the doses of sedative, analgesic, and vasoactive medication. Prior to the skin suturing phase, 50 mg of flurbiprofen was administered to manage postoperative pain.

### Insulin resistance indicators

Peripheral venous blood samples were taken the day before surgery (T1), 10 min prior to anesthesia (T2), and 1 h after surgery (T3) to measure the serum concentrations of fasting glucose (FG), fasting insulin (FINS), and glucagon. A GLM-73 blood glucose analyzer was used to gauge FG concentrations and a double antibody sandwich enzyme-linked immunosorbent assay to determine FINS and glucagon concentrations. The homeostatic model assessment (HOMA) is a widely recognized tool in clinical practice for evaluating fasting-related indicators linked to insulin resistance. The insulin resistance index was calculated using the formula HOMA-IR = FG × FINS/22.5. The insulin secretion index was determined as HOMA-β = 20 × FINS/(FG − 3.5) × 100%, and the insulin sensitivity index was derived as HOMA-IS = 1/(LogFINS + LogFG). In these formulas, the FG unit was millimoles per liter (mmol/L), while the FINS unit was international units per liter (IU/L).

### Gastric ultrasonography assessment

**Fig. 1:**
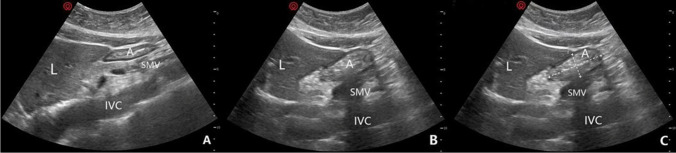
Ultrasonographic images of the gastric antrum. Images in the supine position (**A**). Images in the right lateral decubitus position (RLDP) (**B**). Anterior-posterior (AP) and craniocaudal (CC) diameters of the gastric antrum were measured in the RLDP to calculate antral cross-sectional area (**C**). A = antrum, IVC = inferior vena cava, L = liver, SMV = superior mesenteric vein

GV was checked 10 min before the induction of general anesthesia by employing a bedside gastric ultrasound technique operated by an experienced anesthesiologist, who was unaware of the groupings. Using a low-frequency (2–5 MHz) curved transducer, patients were thoroughly examined in both the supine and right lateral decubitus positions (RLDPs) [17]. The gastric antrum was pinpointed in the sagittal or parasagittal plane, situated between the left liver lobe and pancreas and aligned with the aorta or inferior vena cava (see Fig. 1). In the RLDP, we measured the anterior-posterior (AP) and craniocaudal (CC) diameters. The antral cross-sectional area (CSA) was determined using the formula: CSA = π × (AP × CC ÷ 4). GV in the RLDP was calculated using the following equation: GV (mL) = 27.0 + 14.6 × CSA (cm^2^) − 1.28 × age (a formula attributed to Perlas and colleagues for RLDP) [18]. By further dividing the GV by the patient’s weight, both the GV/W and the occurrence of GV/W > 1.5 mL/kg were assessed. A stomach was deemed “empty” if it either lacked content or held less than 1.5 mL/kg of clear fluid. On the other hand, a “full” designation was given if the stomach had solid content or more than 1.5 mL/kg of clear fluid [19].

Based on the Perlas three-tier semi-quantitative grading system, antrum observations were classified as grade 0 for an empty antrum, grade 1 for minimal fluid only in RLDP, and grade 2 for antrum distention in both the supine position and the RLDP [17].

### Subjective comfort indicators and hemodynamic indicators

The visual analog scale (VAS) was used to gauge subjective comfort indicators such as thirst, hunger, anxiety, fatigue, and nausea for all three groups at T2 and T3. Additionally, the mean arterial pressure and heart rate at various stages were recorded, namely, at admission, after induction, skin incision, pneumoperitoneum, and during laparoscopy.

### Statistical analysis

All statistical analyses were conducted using SPSS version 25.0 (IBM, Armonk, NY, US). The normality of numerical data was assessed using the Kolmogorov-Smirnov test. Data fitting a normal distribution are presented as the mean ± standard deviation. Group differences were analyzed using one-way ANOVA, with subsequent pairwise comparisons using the Bonferroni test. Non-normally distributed data are given as the median (interquartile range) and were analyzed using the Kruskal-Wallis test, with pairwise comparisons made using the Mann-Whitney *U* test. Categorical data are presented as numbers (%) and were analyzed using the *χ*^2^ test or, when appropriate, Fisher’s exact test.

The sample size was determined using PASS 2021 software. Preliminary data indicated an insulin resistance index of 8.0 ± 1.1 in the control group 1 h after surgery. The objective was to identify an insulin resistance difference > 0.8 between the control and carbohydrate groups. Given α = 0.017 and β = 0.2, at least 41 patients were required in each group. Factoring in an anticipated 10% dropout rate, 46 patients were included in each group.

## Results

**Fig. 2:**
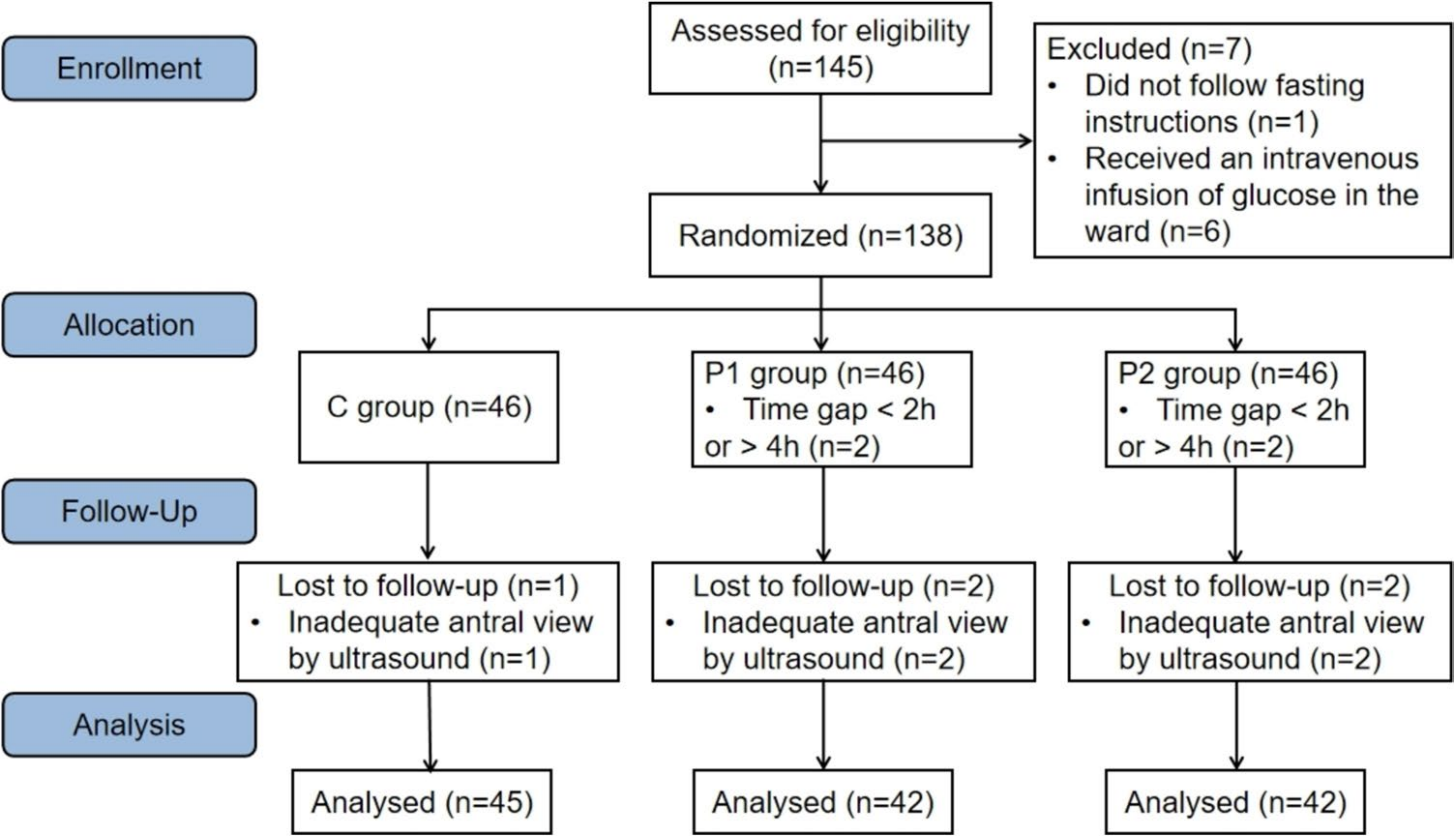
CONSORT diagram

From December 2022 to June 2023, 145 patients were assessed for eligibility to be included in this study. However, 16 were excluded from the final analysis for various reasons: 1 patient did not follow the fasting instructions, 6 received an intravenous infusion of glucose in the ward, 4 took carbohydrates improperly due to temporary changes in the operation time, and 5 did not show clear antral imaging. Ultimately, 129 patients were included in the statistical analysis, 45 in the C group, and 42 in both the P1 and P2 groups. The study protocols are shown in the CONSORT diagram (Fig. 2).

The demographics and surgery-related data of the study population are presented in Table 1. Patient characteristics, operative time, infusion volume, and blood loss were comparable among the 3 groups. Actual fasting for solids and liquids was about 18 h and 16 h, far longer than the stipulated time in the trial guidelines. The time gap between the intake of carbohydrates and tracheal intubation was 161 min for the P1 group and 168 min for the P2 group. These groups showed no notable disparities.

**Table 1:** Comparison of the demographics and surgery-related data of the patients in all groups

	C group (*n* = 45)	P1 group (*n* = 42)	P2 group (*n* = 42)	*p*
Age (y)	49.0 ± 12.3	49.0 ± 13.0	46.4 ± 11.3	0.537
Male sex	20 (44.4)	10 (23.8)	12 (28.6)	0.097
BMI (kg/m^2^)	24.7 ± 3.1	25.1 ± 2.7	24.9 ± 2.5	0.817
ASA score(I/II)	36 (80.0)/9 (20.0)	32 (76.2)/10 (23.8)	38 (90.5)/4 (9.5)	0.207
Diabetes	2 (4.4)	3 (7.1)	1 (2.4)	0.583
Fasting hours for solids (h)	18.0 ± 3.4	17.4 ± 3.1	17.5 ± 2.5	0.603
Fasting hours for liquids (h)	16.0 ± 3.9	15.8 ± 3.3	15.5 ± 2.7	0.783
Time gap (min)	–	161.1 ± 33.9	168.1 ± 36.9	0.367
Duration of surgery (min)	58.0 ± 17.6	57.0 ± 15.5	58.1 ± 14.4	0.943
Infusion volume (mL)	713.3 ± 159.3	665.5 ± 184.6	675.0 ± 130.8	0.335
Amount of bleeding (mL)	8.8 ± 3.5	9.0 ± 3.5	8.3 ± 2.7	0.639

Values are mean ± standard deviation, median (interquartile range), or number (%). Time gap: the actual time gap between preoperative oral carbohydrate and tracheal intubation. *p*: overall comparison

*C group* control group, *P1 group* 200 mL oral carbohydrate group, *P2 group* 400 mL oral carbohydrate group

Baseline measurements for blood glucose, insulin, glucagon, HOMA-IR, HOMA-IS, and HOMA-β were consistent across all three groups. Blood glucose concentrations in the C group were below those in the P2 group 10 min prior to intubation (see Table 2). Other comparisons between groups at T2 and T3 did not reveal any statistically significant differences.

**Table 2:** Insulin resistance indicators in all groups

	C group (*n* = 45)	P1 group (*n* = 42)	P2 group (*n* = 42)	*p*
FPG (mmol/L, mean ± SD)				
T1	5.09 ± 0.63	5.04 ± 0.55	4.96 ± 0.69	0.591
T2	4.73 ± 0.64	5.12 ± 0.80	5.26 ± 1.02^a^	0.011
T3	5.21 ± 0.80	5.30 ± 0.81	5.21 ± 0.85	0.847
FINS (mU/L, mean ± SD)				
T1	39.50 ± 8.54	35.89 ± 9.57	37.25 ± 9.44	0.181
T2	44.01 ± 9.74	44.51 ± 8.50	44.80 ± 8.96	0.920
T3	33.50 ± 7.38	30.48 ± 7.18	32.92 ± 7.47	0.134
Glucagon (pg/mL, mean ± SD)				
T1	376.41 ± 86.07	379.76 ± 70.86	396.32 ± 76.11	0.453
T2	327.88 ± 102.03	300.27 ± 100.36	305.49 ± 96.05	0.389
T3	352.58 ± 114.47	324.07 ± 121.49	362.78 ± 103.25	0.271
HOMA-IR (mean ± SD)				
T1	8.97 ± 2.37	8.13 ± 2.62	8.18 ± 2.25	0.189
T2	9.29 ± 2.52	10.12 ± 2.44	10.38 ± 2.63	0.113
T3	7.78 ± 2.23	7.23 ± 2.22	7.62 ± 2.14	0.489
HOMA-β (median (interquartile range))				
T1	534.65 (361.18)	472.37 (159.49)	526.12 (390.59)	0.266
T2	639.37 (632.64)	590.45 (612.96)	519.74 (682.87)	0.403
T3	389.63 (312.96)	344.07 (257.34)	380.43 (208.78)	0.531
HOMA-IS (median (interquartile range))				
T1	0.28 (0.01)	0.28 (0.02)	0.28 (0.02)	0.246
T2	0.28 (0.01)	0.28 (0.01)	0.28 (0.01)	0.218
T3	0.29 (0.02)	0.29 (0.02)	0.29 (0.01)	0.421

Values are mean ± standard deviation or median (interquartile range). *p*: overall comparison

*T1* preoperative day 1, *T2* 10 min before intubation, *T3* 1 hour after surgery, *FPG* fasting plasma glucose, *FINS* fasting insulin, *HOMA-IR* homeostasis model assessment-insulin resistance index, *HOMA-β* homeostasis model assessment-β, *HOMA-IS* homeostasis model assessment-insulin sensitivity index, *SD* standard deviation

^a^Compared with the C group, *p* < 0.05

The measured gastric antral CSA in RLDP, GV, and the Perlas grade values assessed by gastric ultrasound are presented in Table 3. The mean antral CSAs in the RLDP were significantly greater in the P2 group than in the C group (6.16 ± 0.57 vs. 5.81 ± 0.57 cm^2^, *p* < 0.05) (Table 3). The Perlas grading score revealed a higher frequency of grade 1 (flat antrum in the supine position and the presence of fluid in the RLDP) in the P2 group compared to the P1 and C groups (18 [42.9%] vs. 6 [14.3%] and 1 [2.2%] patients, *p* < 0.05). No Perlas’ grade 2 was found in any of the patients. No statistical differences were detected in GV, GV/W, and GV/W > 1.5 mL/kg among the three groups. None of the patients experienced regurgitation and aspiration.

**Table 3:** Gastric antral CSA, gastric volume, and Perlas grade assessed by gastric ultrasound

	C group (*n* = 45)	P1 group (*n* = 42)	P2 group (*n* = 42)	*p*
CSA, RLDP (cm^2^)	5.81 ± 0.57	6.04 ± 0.66	6.16 ± 0.57^a^	0.024
GV (mL)	49.15 ± 15.14	52.40 ± 17.26	57.51 ± 16.43	0.059
GV/W (mL/kg)	0.74 ± 0.29	0.78 ± 0.26	0.88 ± 0.26	0.057
Perlas 0/1	44 (97.8)/1 (2.2)	36 (85.7)/6 (14.3)	24 (57.1)/18 (42.9)^ab^	< 0.001
GV/W (> 1.5 mL/kg)	1 (2.2)	1 (2.4)	1 (2.4)	0.998

Values are mean ± standard deviation or number (%). *p*: overall comparison

*CSA* cross-sectional area, *GV* gastric volume, *GV/W* gastric volume/weight, *RLDP* right lateral decubitus position

^a^Compared with the C group, *p* < 0.05

^b^Compared with the P1 group, *p* < 0.05

**Fig. 3:**
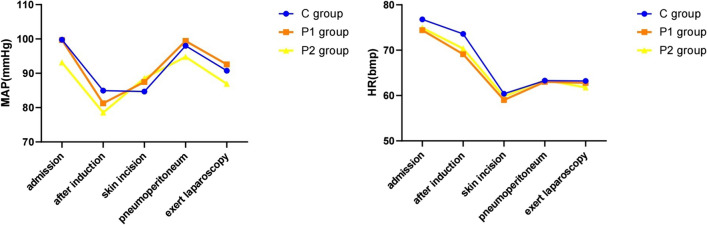
Heart rate and mean arterial pressure

Table 4 shows the VAS scores for subjective comfort factors, including thirst, hunger, anxiety, fatigue, and nausea. The P2 group had notably reduced thirst and hunger scores compared to the C group at both 10-min pre-surgery and 1-h after-surgery intervals. The P1 hunger scores in the P1 group were significantly diminished compared to the C group at T2 and T3. Additionally, hunger scores in the P2 group at T3 were less than in the P1 group. No significant differences in heart rate and mean arterial pressure were found across the three groups (Fig. 3).

**Table 4:** Subjective comfort parameters in all groups

	C group (*n* = 45)	P1 group (*n* = 42)	P2 group (*n* = 42)	*p*
Thirst				
T2	2 (4)	2 (4)	0 (2)^a^	0.001
T3	4 (3)	3 (3)	1.5 (3)^a^	0.001
Hunger				
T2	1 (4)	0 (2)^a^	0 (1)^a^	< 0.001
T3	1 (2)	0 (1)^a^	0 (0)^ab^	< 0.001
Anxiety				
T2	0 (1)	1 (2)	1 (2)	0.178
T3	0 (0)	0 (0)	0 (0)	0.869
Fatigue				
T2	0 (0)	0 (0)	0 (0)	0.217
T3	0 (0)	0 (0)	0 (0)	0.201
Nausea				
T2	0 (0)	0 (0)	0 (0)	0.391
T3	0 (0)	0 (0)	0 (0)	0.306

Values are median (interquartile range). *p*: overall comparison

^a^Compared with the C group, *p* < 0.05

^b^Compared with the P1 group, *p* < 0.05

*T2 *10 min before intubation, *T3 *1 hour after surgery

## Discussion

During this forward-looking randomized controlled study, it was discovered that administering either 200 mL or 400 mL of carbohydrate drink 2–4 h preoperatively had no notable impact on insulin resistance in patients undergoing laparoscopic cholecystectomy. Importantly, this did not adversely affect the risk of pulmonary aspiration relative to traditional midnight fasting. Thus, both carbohydrate intake doses can be used safely for laparoscopic cholecystectomy patients. Consuming 400 mL of carbohydrates effectively alleviated thirst and hunger, whereas 200 mL failed to improve thirst, with neither dosage offering hemodynamic benefits. When selecting the carbohydrate dose, patients should weigh their individual requirements, such as thirst level, cost budget, and night rest.

In prominent tertiary hospitals throughout China, general surgeons often prioritize liver and pancreas surgery during the morning, relegating cholecystectomy procedures to afternoon or evening slots. Regardless of the scheduled time, all patients are routinely instructed to commence fasting from 10 p.m. and to abstain from water intake from 12 p.m. Consequently, patients in our study underwent fasting durations extending up to 18 h. Such protracted fasting intervals can exacerbate preoperative thirst and hunger, intensify perioperative insulin resistance leading to hyperglycemia, elevate postoperative infection rates, and extend hospital stays [4]. Notably, guidelines from the ASA advocate for the consumption of carbohydrate-rich liquids 2–4 h prior to procedures that necessitate general anesthesia [20]. Minimizing the preoperative fasting window can moderate the body’s stress reactions and mitigate insulin resistance induced by prolonged hunger and surgical trauma [5]. Yet deeply entrenched beliefs regarding fasting often present barriers to translating these recommendations into routine clinical practice [21]. There is currently no standardized protocol for preoperative carbohydrate administration, and there is a paucity of clinical trials that have investigated the effects of varying formulations, dosages, and intake methods on postoperative outcomes. Given these facts, there is an urgent need for more research on POC. One study reported that taking oral carbohydrates the evening prior to surgery did not enhance the clinical benefits of consuming them 2–3 h pre-surgery regarding insulin resistance and patient comfort. Moreover, it might disturb the patient’s nighttime sleep [22]. Therefore, in our study, we omitted the evening carbohydrates and assessed only two different carbohydrate dosages taken before surgery.

Post-surgical stress leads to insulin resistance, which manifests as a reduced responsiveness of target organs, tissues, or cells to the actions of insulin [23]. This stress results in the attenuation of insulin’s efficacy in the body and a compensatory rise in insulin secretion, concomitantly elevating the blood glucose concentration [24]. The peak of this phenomenon typically manifests on the day of surgery. Introduced by Matthews et al. in 1985, HOMA serves as a mathematical representation, encapsulating the interplay between glucose and insulin across diverse organs [25]. This model evaluates both insulin resistance and the performance of existing β cells using just fasting blood glucose and insulin measurements [26].

Paradoxically, while numerous investigations utilizing HOMA have unveiled a pronounced decrement in postoperative insulin resistance between carbohydrate and fasting cohorts [27, 28], others have observed no such divergence [29–31]. Onalan et al. reported reduced insulin resistance 3 h post-surgery in patients who received an oral carbohydrate solution, initially 800 mL at midnight before surgery, followed by 400 mL at 6 a.m. on the day of surgery [32]. Yadav et al. noted a decrease in insulin resistance 24 h after administering oral maltodextrin to non-insulin-dependent diabetic patients [28]. These findings differ from ours, possibly because our oral solution was consumed in a single dose 2 h before surgery, not the night before. Also, our study was not confined to diabetic patients. Pędziwiatr et al. found no significant difference in insulin resistance between groups, aligning with our results [31]. However, their study had a smaller sample size of only 40 patients and a single intervention group without varying fluid doses. Thus, there’s a pressing need for more uniform clinical studies.

Our analysis found that neither 200 mL nor 400 mL of pre-surgical carbohydrate loading markedly altered insulin resistance in patients undergoing laparoscopic cholecystectomy. This observation can be partly attributed to the fact that preoperative oral carbohydrate solution is just one aspect of the enhanced recovery after surgery (ERAS) protocol. Its role in hastening perioperative recovery within the comprehensive ERAS framework is relatively limited, making it challenging to demonstrate differences in accelerated rehabilitation solely based on this measure. Nonetheless, combining preoperative oral carbohydrate with other ERAS protocols such as early postoperative feeding could have a synergistic effect. Early feeding post-surgery is known to reduce catabolism and maintain muscle mass, which can be further enhanced if the body is better prepared metabolically through pre-surgical carbohydrate loading.

In recent years, bedside gastric ultrasonography has emerged as a straightforward, efficient tool empowering anesthesiologists to measure gastric fullness and to appraise pulmonary aspiration risks by visualizing pre-anesthesia gastric contents and volume [33]. In one study, researchers employed gastric ultrasound on patients scheduled for elective laparoscopic benign gynecological surgery and found that a carbohydrate drink consumed 2 h pre-surgery did not amplify residual GV vis-à-vis midnight fasting [14]. Our data revealed amplified CSA measurements in the right lateral decubitus stance for the 400 mL cohort when juxtaposed against the fasting group. The Perlas grade exhibited an increased trend in the 400 mL carbohydrate cohort compared to its 200 mL and fasting values. Notably, there were no differences in GV, GV/W, and GV/W > 1.5 mL/kg among the groups, findings consistent with previous studies [34, 35]. None of the patients had Perlas’ grade 2 on gastric ultrasound examination, indicating that the gastric clearance of carbohydrate beverages was not hindered by POC.

Our research found that carbohydrate beverages significantly allayed sensations of hunger and thirst, corroborating the findings of previous studies [36, 37]. Bisgaard et al. discovered that preoperative oral carbohydrate solution did not alleviate postoperative discomfort, contrasting with our study’s findings [38]. This discrepancy could stem from the fact that their control group received a placebo instead of undergoing overnight fasting. Nevertheless, no discernible disparities were detected in intraoperative hemodynamic shifts between the three cohorts of patients. In contrast to major abdominal surgery, LC is a short and minimally invasive procedure, and therefore, the effects of carbohydrates are notably diminished.

Our study had several limitations. First, our study did not evaluate patients’ insulin resistance and subjective comfort for several hours after surgery. Second, given that cholecystectomy is often not the first surgery of the day, precise surgical commencement times remain unpredictable. This uncertainty complicates the determination of the interval between carbohydrate intake and intubation, leaving us an approximate 2-h margin rather than pinpoint accuracy—yet our findings echo tangible clinical practice. Third, our cohort was restricted to individuals aged 18–65 years old with standard BMI scores, but excluded minors, seniors, and morbidly obese patients. Subsequent studies, expanding the demographic scope, are warranted. Fourth, baseline gastric ultrasonography was omitted. However, patients with potential gastric-emptying delays were excluded, and strict fasting regimens were enforced [39]. Fifth, trials in this area varied in the study population, drinking regimen, control group composition (placebo or fasting), and sample size. Therefore, there is a need for more uniform study designs in future research. Lastly, future studies should encompass expansive, multi-center research initiatives.

## Conclusion

Oral doses of 200 mL or 400 mL of carbohydrates 2–4 h before surgery did not significantly affect insulin resistance or gastric emptying in patients undergoing laparoscopic cholecystectomy. Therefore, both doses can be used safely in laparoscopic cholecystectomy patients. When selecting the carbohydrate dose, patients should weigh their individual requirements.

## Data Availability

The data that support the findings of this study are available from the corresponding author, upon reasonable request.
